# Hook Fabric Electroencephalography Electrode for Brain Activity Measurement without Shaving the Head

**DOI:** 10.3390/polym15183673

**Published:** 2023-09-06

**Authors:** Granch Berhe Tseghai, Benny Malengier, Kinde Anlay Fante, Lieva Van Langenhove

**Affiliations:** 1 Department of Materials, Textiles and Chemical Engineering, Ghent University, 9000 Gent, Belgium; benny.malengier@ugent.be (B.M.); lieva.vanlangenhove@ugent.be (L.V.L.); 2 Jimma Institute of Technology, Jimma University, Jimma P.O. Box 307, Ethiopia; kinde.anlay@ju.edu.et

**Keywords:** brain activity, EEG, e-textile, hook fabric, dry electrode, textrode

## Abstract

In this research, novel electroencephalogram (EEG) electrodes were developed to detect high-quality EEG signals without the requirement of conductive gels, skin treatments, or head shaving. These electrodes were created using electrically conductive hook fabric with a resistance of 1 Ω/sq. The pointed hooks of the conductive fabric establish direct contact with the skin and can penetrate through hair. To ensure excellent contact between the hook fabric electrode and the scalp, a knitted-net EEG bridge cap with a bridging effect was employed. The results showed that the hook fabric electrode exhibited lower skin-to-electrode impedance compared to the dry Ag/AgCl comb electrode. Additionally, it collected high-quality signals on par with the standard wet gold cups and commercial dry Ag/AgCl comb electrodes. Moreover, the hook fabric electrode displayed a higher signal-to-noise ratio (33.6 dB) with a 4.2% advantage over the standard wet gold cup electrode. This innovative electrode design eliminates the need for conductive gel and head shaving, offering enhanced flexibility and lightweight characteristics, making it ideal for integration into textile structures and facilitating convenient long-term monitoring.

## 1. Introduction

**Figure 1:**
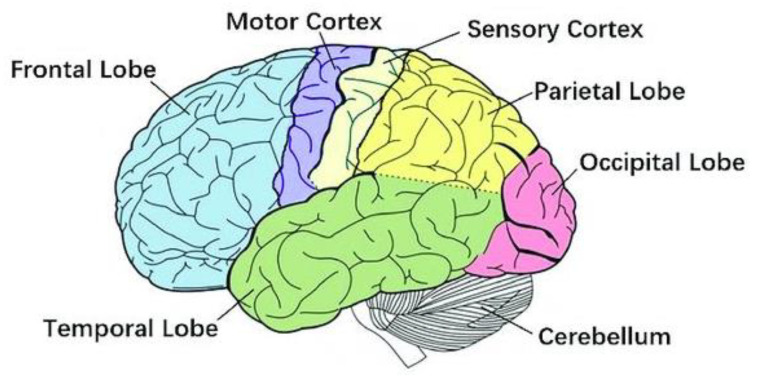
The human brain [1], under CC by 4.0.

The brain, often called the “command center,” is a complex organ comprising over 100 billion nerves communicating through trillions of synapses. It consists of a variety of specialized sections, shown in Figure 1, that collaborate [1].

**Figure 2:**
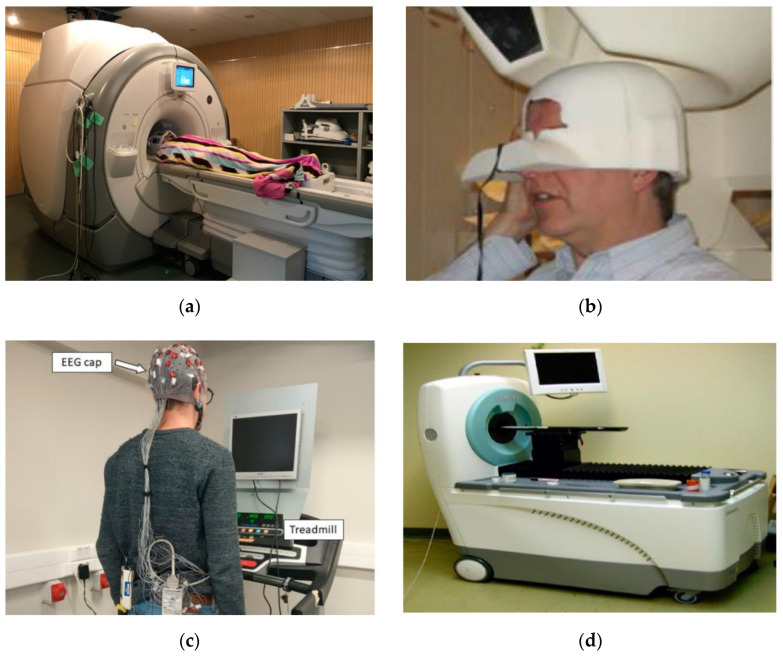
Brain activity measurement approaches: (**a**) Functional Magnetic Resonance Imaging (fMRI) [2], under CC by 4.0; (**b**) Magnetoencephalography (MEG) [3], under CC by 3.0; (**c**) Electroencephalography (EEG) [4], under CC by 4.0; (**d**) Positron Emission Tomography (PET) [5], under CC by 4.0.

The human brain constantly operates as a frequency wave, with brain cells transmitting and receiving electrical signals even during sleep. Functional magnetic resonance imaging (fMRI), magnetoencephalography (MEG), and electroencephalography (EEG) are the three most prevalent and widely used techniques (Figure 2) for measuring brain activity. Among these, EEG is the most adaptable and cost-effective.

Electroencephalography (EEG) is the direct recording of the brain electrical activity of neuronal interactions by placing electrodes on the scalp surface. This widely used brain activity measurement technique offers excellent time resolution of brain activity and also provides cost and space advantages over other technologies like magnetoencephalography, functional magnetic resonance imaging, and positron emission tomography.

EEG is used to diagnose brain-related seizures and inflammation, including epilepsy, Parkinson’s disease [6], and stroke [7]. The application of EEG in brain–computer interfaces [8], sleep disorder diagnosis [9], and cognitive neuroscience [10] is booming.

Silver/silver chloride (Ag/AgCl) and gold cup wet electrodes are widely utilized as standard EEG signal acquisition tools due to their excellent signal quality, reproducibility, and biocompatibility. Despite this, EEG measurements using these electrodes necessitate skin preparation and the application of conductive paste to reduce scalp–electrode contact impedance. This process is time-consuming, requires trained personnel, and can result in neurotic excoriation lesions from skin scratching. Additionally, the use of conductive pastes may lead to hair damage and allergic reactions.

**Figure 3:**
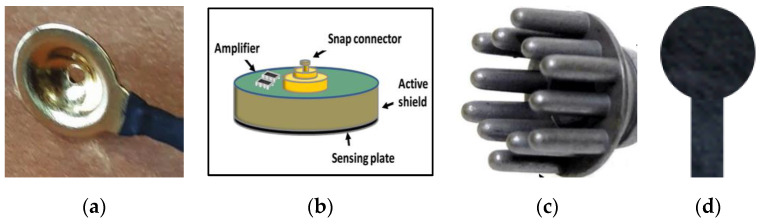
Types of EEG electrodes: (**a**) gold cup EEG electrode [11], under CC by 4.0; (**b**) capacitive EEG electrode [17], under CC by 4.0; (**c**) dry EEG comb electrode [28], under CC by 4.0; (**d**) dry EEG flat electrode [29], under CC by 4.0.

The associated problems with wet Ag/AgCl and gold cups (Figure 3a) led to the development of dry electrodes made of a conductive substance that mechanically interfaces with the skin, avoiding the skin preparation procedure and the use of conductive paste [11]. They can work without any physical contact on pure capacitive coupling (Figure 3b) or with dry sensor–skin contact (Figure 3c,d). Capacitive EEG electrodes [12,13,14,15,16] are unlikely to be appropriate for recording spontaneous EEG signals, as the amplitude levels are so low; moreover, the high impedance of these electrodes, on the other hand, necessitates the use of an ultra-high impedance on-site amplifier [17]. In addition, the “floating” fixation on the scalp causes motion artifacts. Skin-contact dry EEG electrodes were also introduced [18,19,20,21]. Flat skin-contact dry electrodes cannot be used in the hairy regions. Although needle-based dry electrodes, such as a 3D-print legged electrode reported by Tong et al. [22], can be used on the hairy region, they are still heavy in weight and stiff in structure. Mascia et al. have developed a dry tattoo EEG electrode [23]; such electrodes are highly affected by sweating and skin temperature, causing nonevent sensing, and decay shortly with age. Thus, all the aforementioned EEG electrodes are inappropriate for wearable purposes. These limitations have led to the emergence of textile-based electroencephalography (EEG) electrodes. Therefore, in a previous study [24,25,26], we successfully developed dry EEG electrodes from PEDOT:PSS/PDMS-printed cotton fabric [27]. The electrode-collected EEG signals are comparable to those of the dry Ag/AgCl EEG comb electrodes; however, placing such an electrode on the hairy part of the head is not possible because it is a flat electrode. Thus, shaving the head is compulsory prior to the use of this electrode. Moreover, tens or hundreds of electrodes are required to monitor brain activity with EEG electrodes, where any part of the head is shaved each time the measurement is intended because the hair grows rapidly, which in turn increases the skin-to-electrode contact impedance and affects signal quality. Therefore, this trend is unsuitable for long-term monitoring. In addition, shaving the head is an extra burden for experts and time-consuming. As a result, developing a textile-based dry EEG electrode that can be used even in a hairy region would undoubtedly provide a significant advantage in addressing the limitations of existing electrodes. In this study, a dry EEG electrode was made from conductive hook fabric and compared to a dry EEG comb electrode.

In addition, a textile-based knitted-net EEG bridge with comparable performance to a universal EEG bridge was introduced to maintain the electrodes in the required position. The new EEG bridge is 100% textile, which is a technical advantage offering flexibility and low weight over universal EEG bridges.

## 2. Materials and Methods

### 2.1. Textile-Based Electrode (Textrode) Design

**Figure 4:**
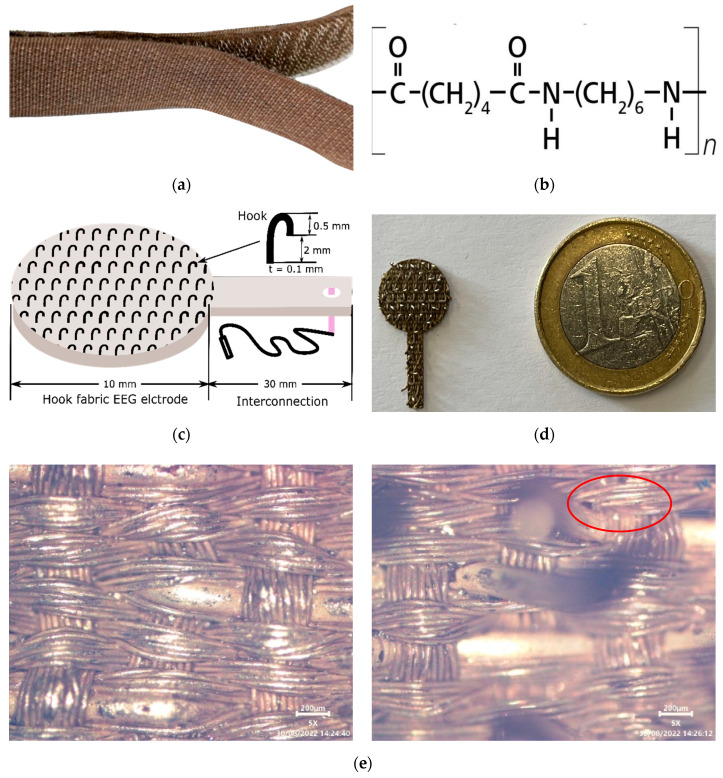
(**a**) Photographic image of Velcro^®^ hook tape; (**b**) chemical structure of DuPont Nylon (Nylon 66); (**c**) schematic illustration of hook fabric textrode; (**d**) actual hook fabric EEG textrode; (**e**) face (**right**) and back (**left**) optical microscopic view of the and EEG textrode with the red circle indicating a hook.

The acronym ‘textrode” was used for the ‘textile electrode’ in this study. A conductive Velcro^®^ tape made of 100% silver-plated DuPont Nylon fiber durable material (Figure 4a) and 10,000 openings/closings high cycle life with a surface resistivity of 1 Ω/cm and greater than 60 dB electromagnetic shielding effectiveness (Light Stitches, Wotton-under-Edge, UK) was used to construct the electrodes. Circular electrodes with a diameter of 1 cm (surface area of π cm^2^) and the same size as the comb Ag/AgCl dry EEG electrode were constructed [30]. A schematic illustration, photographic and microscopic image of the hook fabric electrode are shown in Figure 4.

### 2.2. Skin-to-Electrode Impedance Measurement

**Figure 5:**
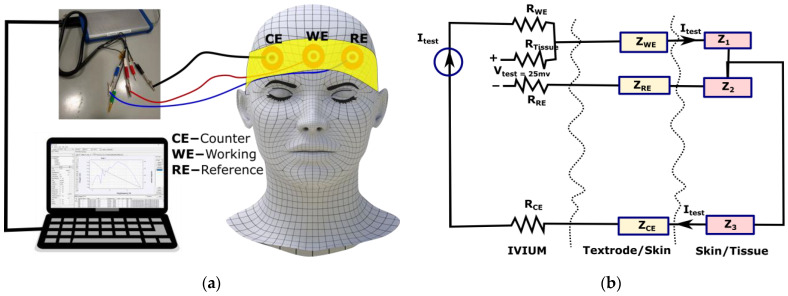
Impedance measurement: (**a**) schematic illustration of three-electrode configuration impedance measurement with IVIUM potentiostat; (**b**) impedance measurement [31], under CC by 4.0.

When electrodes are used to monitor biopotentials, the impedance of the system is composed of the impedances of the electrode and skin. As a result, skin-to-electrode impedance is crucial for collecting high-quality EEG signals. The impedance of the skin-electrode combination was measured using an IVIUM potentiostat setup with a three-electrode configuration according to [15]. For the IVIUM instrument, conventional wet electrodes were employed as counter and reference electrodes. To allow impedance stability at the contact interface, they were positioned 10 cm apart on the skin/phantoms and kept in place for 30 min before starting the measurements. IVIUM’s working and sensing cables of the IVIUM were attached to the electrode under test, which was next to the reference electrode. The hook fabric electrodes were employed as the working electrodes, but a dry Ag/AgCl comb and standard wet gold cup electrodes were also characterized to compare this result with the results of the other hook fabric electrodes. The potentiostat controlled a 25 mV AC signal between the working and reference electrodes during the experiments, with a signal frequency ranging from 0.1 Hz to 60 Hz. The current traveled mostly through the working and counter electrodes, as well as the skin and tissue between them because the internal impedance of the IVIUM’s voltage meter is above 1000 GΩ. In the schematic illustrated in Figure 5, the corresponding impedances are labeled as ZWE (impedance of skin-to-working electrode), ZRE (impedance of skin-to-reference electrode), and ZCE (impedance of skin-to-counter electrode). Z1 represents the impedance of the tissue between WE and RE), Z2 represents the impedance of the tissue between RE and CE, and Z3 represents the impedance of the tissue between WE and CE.

### 2.3. Knitted Net EEG Electrode Bridge

**Figure 6:**
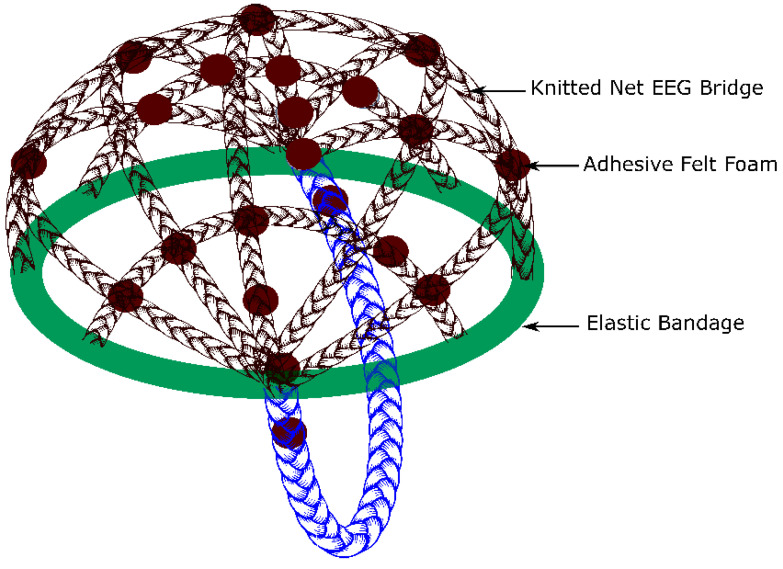
A hand-knitted net EEG bridge (EEG cap).

A multifilament polyester yarn was used to create a knitted net EEG electrode bridge by hand knitting [30]. The knitted net EEG electrode bridge could simultaneously support up to 21 electrodes. Felt foam with a diameter of 13.5 cm was placed on the knitted net bridge. The felt foam exerts pressure on the electrode, preventing it from sliding and allowing for better skin-to-electrode contact. Its side towards the electrode has an adhesive that maintains the electrode in place. In addition, felt foam, which is a permanent part of the bridge, can be used as a guideline for electrode placement using the 10–20 electrode placement system. The knitted net EEG electrode bridge is adjustable, allowing the user to tighten or loosen it, depending on the size and shape of the subject’s head. The positions of the felt foams were also adjustable within the knitted net EEG electrode bridge. A schematic illustration of the knitted net EEG electrode bridge is shown in Figure 6.

### 2.4. EEG Measurement

**Figure 7:**
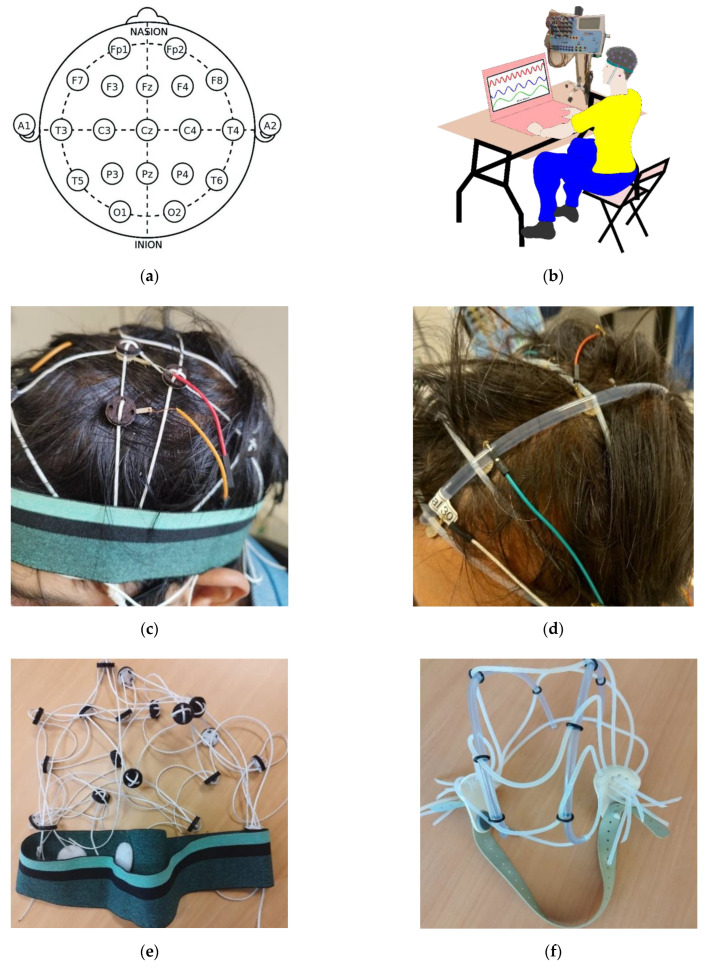
(**a**) The 10–20 EEG placement system; (**b**) schematic illustration of EEG measurement setup; (**c**) photographic image of the EEG measurement at a clinical level using the knitted net EEG electrode bridge; (**d**) photographic image of the EEG measurement at a clinical level using Universal EEG cap; (**e**) photographic image of the knitted net EEG electrode bridge; (**f**) photographic image of the Universal EEG cap.

The EEG measurement was performed at the Department of Neurology in UZ Gent, Ghent University Hospital. Waveforms were recorded using a Brain Quick^®^ Clinical EEG Line [32]; the device supports Micromed–SystemPLUS Evolution 1.4 software. Twelve hook fabric electrodes, 10 active, and two reference electrodes were placed on the hairy region of the head according to the 10–20 EEG placement system, as shown in Figure 7a. The 10 and 20 in the system indicate the distance of the electrodes from each other in proportion to the size of the head. The letters in the indicated positions represent the corresponding position of the head, with even numbers representing the right position and odd numbers the left. The schematic illustration of the EEG measurement setup used in this work is shown in Figure 7b.

The knitted net EEG electrode bridge/EEG cap (Figure 6) was used to keep the electrodes in the required positions according to the 10–20 system, preventing them from sliding. The EEG cap has non-conductive foams placed on top of the electrodes to ensure the electrode is sufficiently pressed against the skin. The performance of the knitted net EEG electrode bridge was compared with a universal EEG cap [33]. Figures 7c and 7d are photographic images of the EEG measurement at the clinical level using the hook fabric EEG textrodes. Figures 7e and 7f are photographic images of the knitted net EEG electrode bridge and universal EEG cap, respectively.

The collected signals were also analyzed using EEGLab (version 2021.1) software. A Florida Research Instrument Inc reusable Ag/AgCl dry EEG comb electrode (TDE-20-15 reusable EEG electrode) [34] was obtained from OpenBCI.

### 2.5. ITC, ERSP and PSD Analysis

An EEGLAB (version 2021.1) software [35] was used to perform data treatment and statistics offline. The EEGLAB allows for the generation of time-frequency images and for calculating event-related spectral perturbation (ERSP) and intertrial coherence (ITC). ERSP measures how much the power at various frequencies in signal changes in proportion to a certain time point [36], whereas ITC measures the consistency of the oscillatory phase throughout a set of trials [37]. A 250 Hz low-pass filter, a 256 Hz sampling rate, and a 0.5 Hz high-pass filter were used in the beginning. ERSP was obtained for the time-domain analysis by averaging baseline-corrected epochs taken from 0 to 9.996 s after the target apparition event. From the initial 2560 epochs (5% discarded), a total of 2432 epochs remained after artifact rejection. Meanwhile, ITC was obtained for wave cycles from 3 to 0.5, epoch time limit from 0 to 9.996, and frequency limit from 0.5 to 250 Hz. The power spectral density (PSD) of the textrode was also compared to wet gold cups and dry Ag/AgCl electrodes.

### 2.6. Signal to Noise Ratio (SNR) Analysis

**Figure 8:**
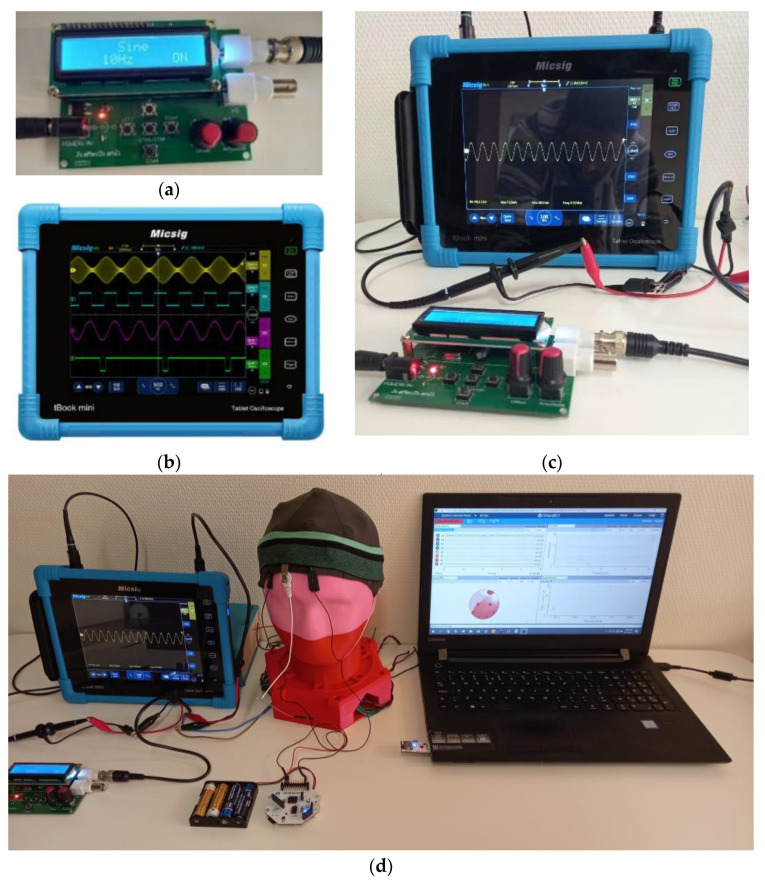
Synthetic sine wave generation: (**a**) function generator; (**b**) digital oscilloscope; (**c**) synthetic sine wave generated by the function generator on a digital oscilloscope; and (**d**) injecting synthetic EEG wave in to head phantom and measuring back with OpenBCI board.

For the SNR analysis, a single active electrode was used. A function generator (Figure 8a) and Micsig TO1104 Handheld Tablet Digital Oscilloscope (Figure 8b) were used to generate a synthetic sine wave (360 mV peak to peak voltage, 9.925 Hz frequency, 50 ms time). The generated synthetic sine wave (Figure 8c) was then injected into a textile-based head phantom (Figure 8d). To impersonate events according to [38], the EEG phantom signal parameters were set in the alpha wave range and the amplitude was varied from 0.2 to 1 V to mimic a neurological event. Then, the EEG wave was compared to waves from a dry Ag/AgCl comb electrode under the same conditions using an OpenBCI board. Next, the EEG signals were denoised using EEGLAB. Finally, the quality of signals collected was analyzed mathematically in terms of signal-to-noise Ratio (SNR) using Equation(1).

SNR (dB) = 20log(Peak to Peak Voltage Signal)/(Peak to Peak Voltage Noise)(1)

Additionally, the impact of electrode size and bending on the SNR were assessed. Initially, the dimensions of the hook fabric EEG electrode were matched to the commercial dry Ag/AgCl comb electrode size (surface area of π cm^2^) to ensure a fair comparison. Then, the effect of doubling the electrode size (surface area of 2π cm^2^) was studied and subsequently it was increased fourfold.

To investigate the effects of bending, the commercial dry Ag/AgCl comb electrode size hook fabric electrode was subjected to up to 40 bending cycles with a 5 mm radius bending. The result was analyzed using a one-way ANOVA at a 95% confidence interval.

## 3. Results and Discussions

### 3.1. Performance of the Knitted Net Bridge EEG Cap

**Figure 9:**
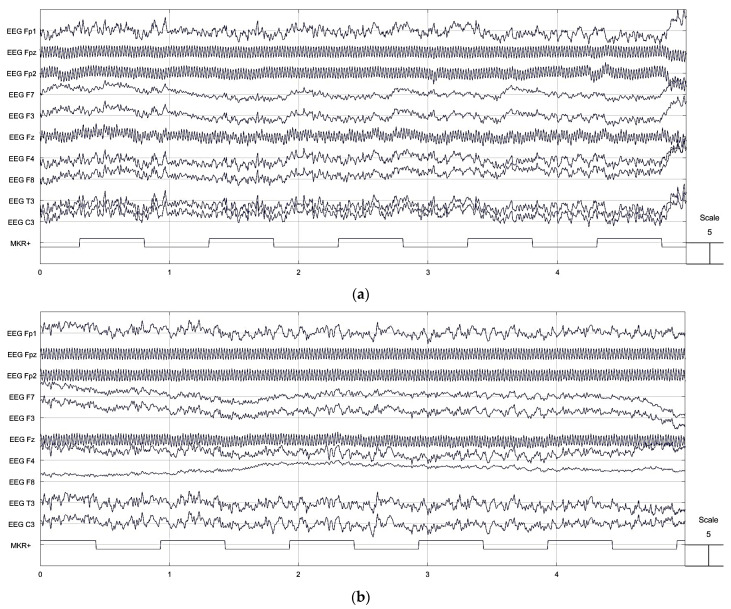
EEG channel data from (**a**) Universal EEG bridge; (**b**) knitted net bridge EEG cap.

The EEG channel data collected by dry Ag/AgCl comb electrodes using a universal EEG cap and the knitted net bridge EEG cap are equivalent, as shown in Figure 9. Fpz, Fp2, and Fz were kept railed in both EEG bridges, and the effect appeared in the knitted net bridge EEG cap similar to the universal EEG cap. This shows that the knitted net bridge EEG cap can be used in its present form at the clinical level as a replacement for currently used caps.

**Figure 10:**
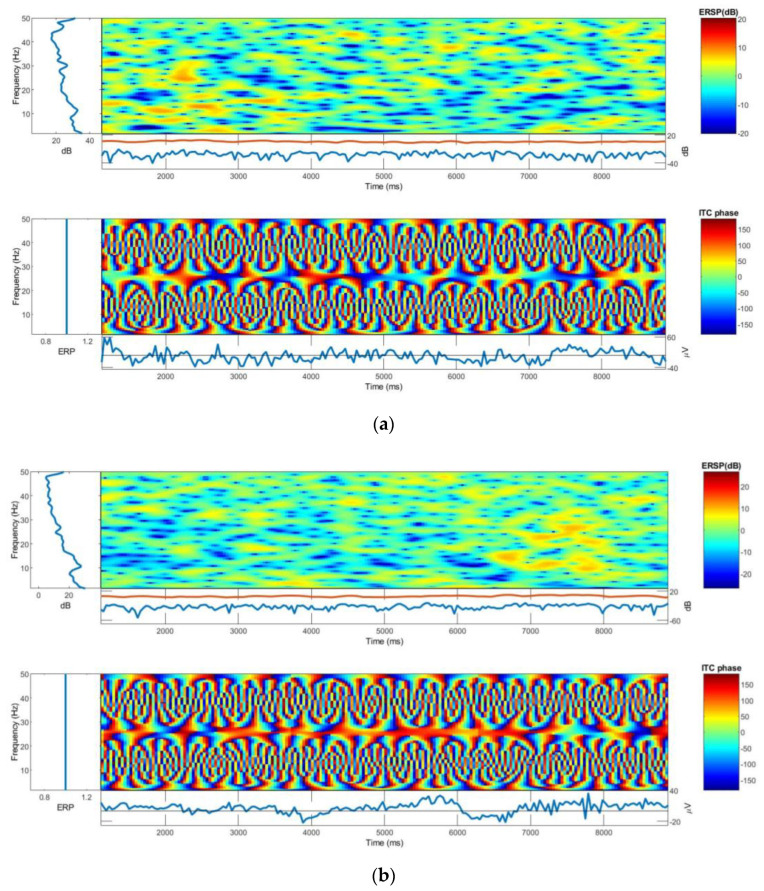
ERSP and ITC Phases: (**a**) Universal EEG bridge; (**b**) knitted net bridge EEG cap.

The output graph of ERSP and ITC EEG signals collected by the knitted net bridge EEG cap and universal EEG cap, shown in Figure 10, were compared using EEGLAB (version 2021.1) software. In each plot, the frequency range and the time range are placed on the *y*-axis and *x*-axis, respectively, and a color scale is used where green is for non-significant and red represents significant ITC at a 99% CI. Beneath each ITC plot is the averaged ERSP response for that individual (in blue), in microvolts.

The amplitude scale for the ERSP response is equivalent for both the knitted net bridge EEG cap and universal EEG cap with a maximum value of 99 µV. To the left of each ITC plot, the average power is shown for that electrode at each frequency, while a black dotted line shows the significance threshold at each frequency relative to the baseline period at a 95% confidence level. The ERSP and ITC phases, and channel time frequencies for Fp1, of both bridges are shown in Figure 10. From the EEGLAB (version 2021.1) software analysis, the maximum log power distribution for both bridges also equally ranged from −50 to 50 dB. The maximum log PSD for both electrodes was ∼40 µV^2^/Hz. Therefore, the knitted net bridge EEG cap can replace the universal EEG cap providing weight, price, and accessibility advantages.

### 3.2. Skin-to-Electrode Contact Impedance Comparison

**Figure 11:**
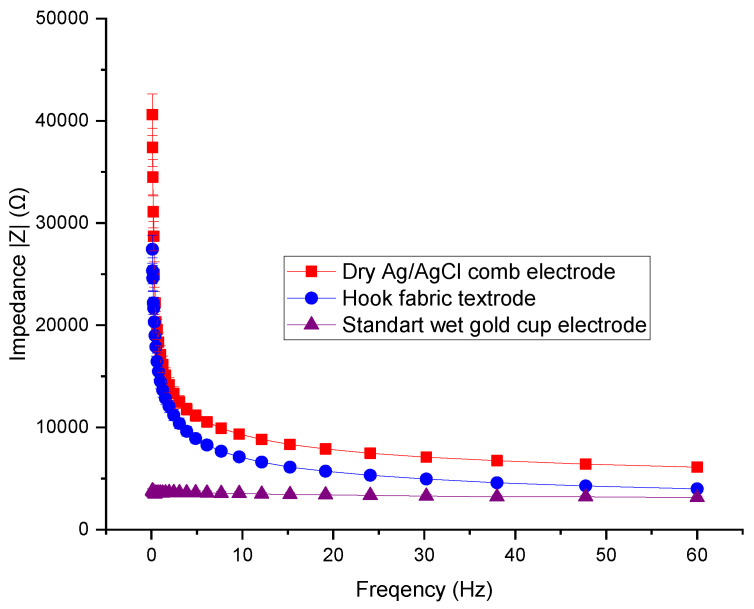
Skin-to-electrode impedance from IVIUM Potentiostat.

Throughout all frequencies, the skin-to-electrode contact impedance of the hook fabric textrode was significantly lower than the dry Ag/AgCl comb electrode, as shown in Figure 11. The one-way ANOVA at a 95% CI showed the skin-to-electrode contact impedance of the hook fabric textrode and dry Ag/AgCl comb electrode is significantly different, providing an *f*-ratio value of 2.81 and *p*-value of 0.01. These results could be because the hook fabric textrode has more contact points than the dry Ag/AgCl comb electrode. Thus, the hook fabric textrode could have a technical advantage over the dry Ag/AgCl comb electrode EEG monitoring, especially for wearable applications, as the impedance is lower.

At alpha, beta, and gamma frequencies, the skin-to-electrode contact impedance of the hook fabric texture was lower than that of the dry Ag/AgCl comb electrode, as shown in Figure 11. The one-way ANOVA at a 95% CI showed that the skin-to-electrode contact impedance of the hook fabric textile and dry Ag/AgCl comb electrode was significantly different, providing an *f*-ratio value of 2.81 and a *p*-value of 0.01. These results could be because the hook fabric electrode has more contact points than the dry Ag/AgCl comb electrode. Thus, the hook fabric textrode could have a technical advantage over dry Ag/AgCl comb electrode EEG monitoring, particularly for wearable applications. However, the impedance was higher than that of standard wet gold cup electrodes, which agrees with the existing literature that states that wet electrodes provide lower skin-to-electrode impedance than commercial dry electrodes [39]. However, the impedance increases with time due to dehydration, even at room temperature, as a conductive gel or electrolyte is employed to lower the impedance. Therefore, hook fabric textrodes are potential candidates for the detection of cardiac and neural activity.

### 3.3. EEG Signal Comparison

**Figure 12:**
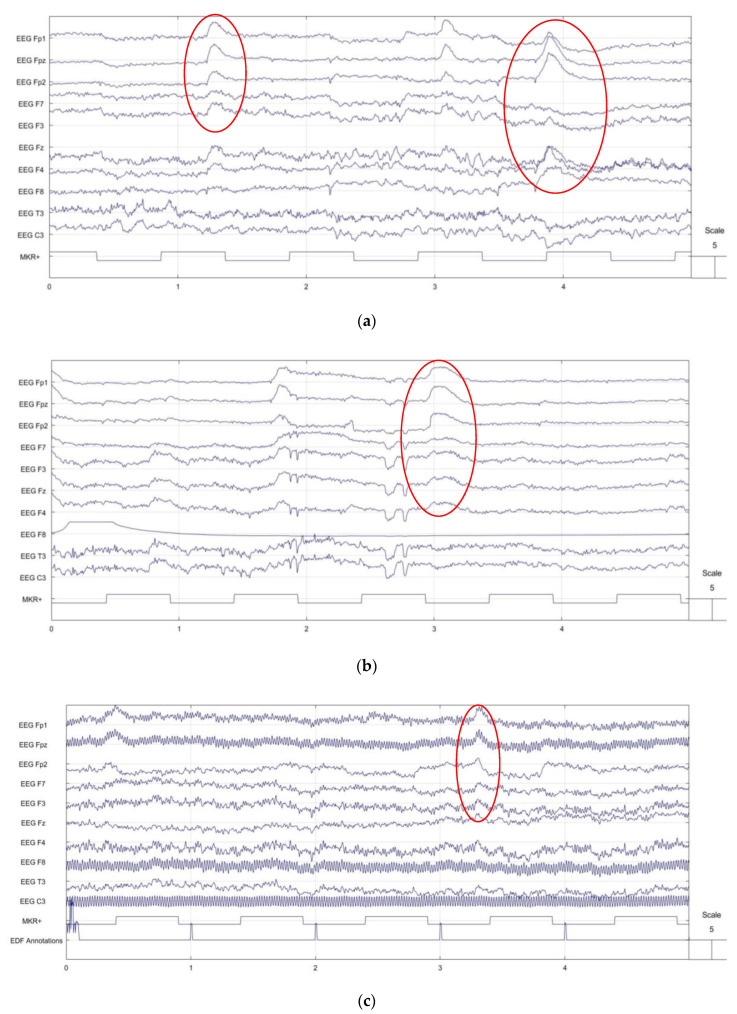
EEG signals using knitted net EEG bridge: (**a**) hook fabric textrode; (**b**) dry Ag/AgCl comb electrode; (**c**) standard wet gold cup electrode.

The EEG signals recorded by the textrode were comparable to those of standard gold cups and dry Ag/AgCl comb electrodes. The amplitude of the signal was as high as that of the standard wet gold cup and dry Ag/AgCl comb electrodes in all head regions. In addition, the textrode provides a similar bandwidth range. The EEG signals from the textrode, standard wet gold cup, and dry Ag/AgCl comb electrodes from a healthy subject with no history of brain health problems are shown in Figure 12. In addition, blinking and mouth-opening artifacts were clearly recognized and identified in the novel hook fabric textrode in the same manner as in the standard wet gold cup and dry Ag/AgCl comb electrodes, as marked in red in the images in Figure 12. This textrode is, therefore, a promising alternative to the existing EEG electrodes, offering weight, flexibility, and economic benefits. The EEG signals collected with the hook fabric textrode had a similar EEG pattern to those collected by the standard wet gold cup and dry Ag/AgCl comb EEG electrodes. When comparing the images, there is no doubt that the EEG waves from a human cannot be exactly identical, even within the same electrode, as the human brain cannot be in a constant state. Hence, for commercial use, these electrodes should be validated using head phantoms.

### 3.4. ITC, ERSP and PSD Analysis

The European Data Format (EDF) file types of the EEG waves in Figure 12 were analyzed using EEGLAB (version 2021.1) software. The ITC, ERSP, and PSD graphs generated from the hook fabric, dry Ag/AgCl, and wet gold cup electrodes are discussed below.

#### 3.4.1. ERSP

**Figure 13:**
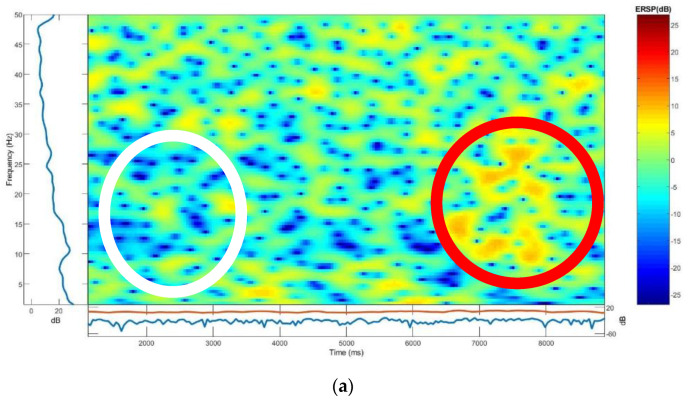

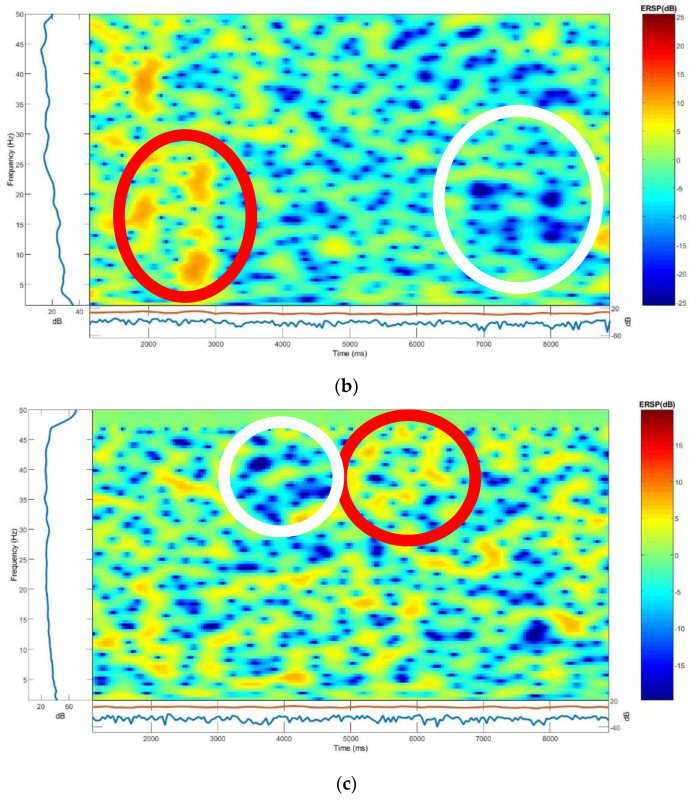
ERSP phases: (**a**) hook fabric textrode; (**b**) dry Ag/AgCl comb electrode; (**c**) standard wet gold cup electrode.

Figure 13 shows mean event-related changes in spectral power at each time during the epoch and at each frequency, <50 Hz. The amplitude scale for the ERSP response is 20 dB to 60 dB and similar for hook fabric textrode, dry Ag/AgCl comb, and wet gold cup electrodes. As marked with white and red on the images in Figure 13, the spectral power was found to be fairly similar in both the dry Ag/AgCl and the hook fabric electrodes across the frequency domain. For both, the ERSP envelope, low and high mean dB values relative to baseline were ∼17 dB at any time in the epoch. Visual observation shows that green, yellow and blue are evenly distributed over the region and are similar for both electrodes.

#### 3.4.2. ITC

**Figure 14:**
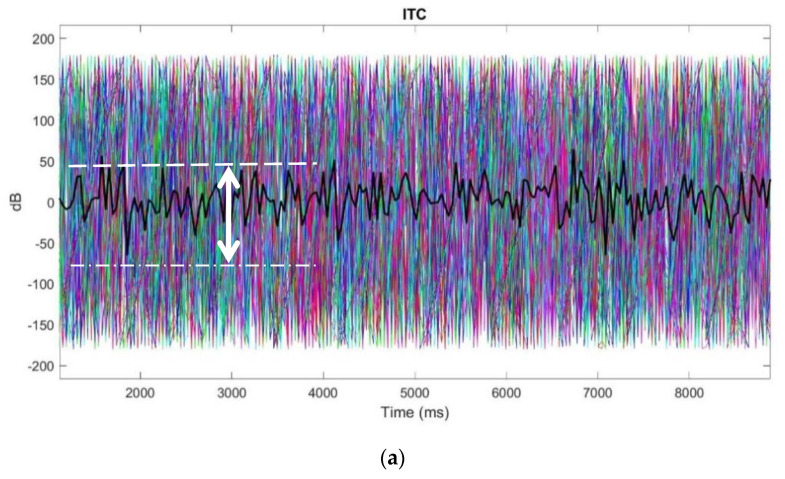

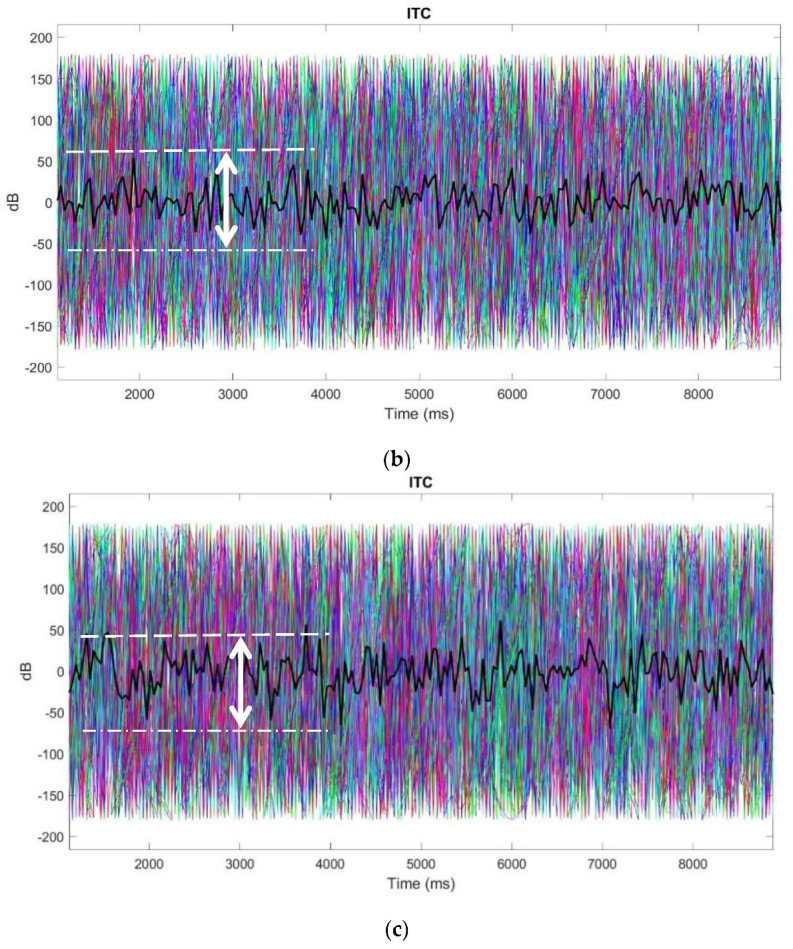
ITC phases: (**a**) hook fabric textrode; (**b**) dry Ag/AgCl comb electrode; (**c**) standard wet gold cup electrode.

Figure 14 shows the ITC phases of the hook fabric test, standard wet gold cup, and dry Ag/AgCl comb electrodes at all frequencies. In the ITC, the ratio of the spectral power distribution similarly ranges between −50 and 50 dB, as marked with a white arrow in the images in Figure 14. Most importantly, the ITC plots were equivalent. Therefore, the ITC plots prove that the hook fabric EEG textrode can be used without a doubt to monitor brain activity in its present form, even at a clinical level. Further design improvements would, of course, make the textrode a better choice than existing electrodes for the proposed application.

#### 3.4.3. PSD

**Figure 15:**
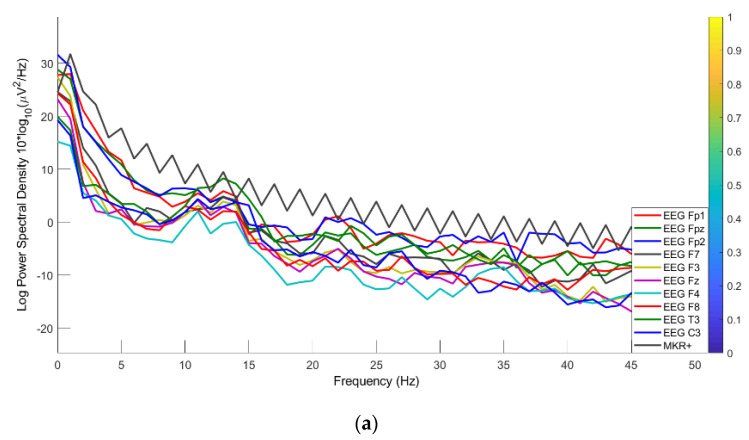

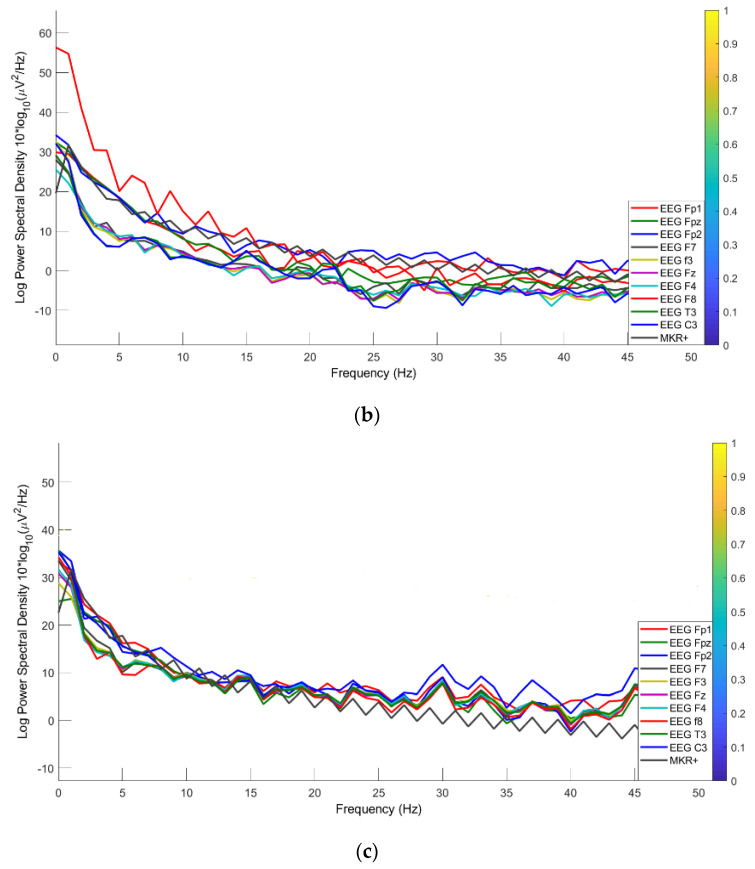
Channel spectra: (**a**) hook fabric textrode; (**b**) dry Ag/AgCl comb; (**c**) standard wet gold cup electrodes.

The PSD representing the power distribution of EEG series in the frequency domain was also used to evaluate the hook fabric EEG textrode against standard wet gold cup and dry Ag/AgCl comb electrodes across 0.1 to 50 Hz covering all EEG bandwidths. Specifically, the log PSD decreased linearly with an increase in the frequency domain, and the maximum log PSD for all electrodes was in the delta EEG bandwidth of ∼35 µV^2^/Hz, as shown in Figure 15. Therefore, the hook fabric EEG textrode can be used exactly in its present form, as per this comparison.

### 3.5. Signal-to-Noise Ratio (SNR)

**Figure 16:**
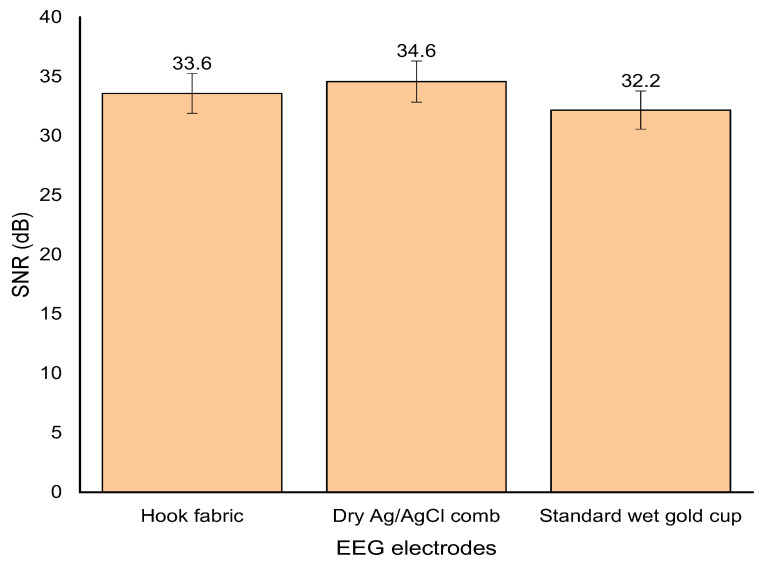
Signal-to-noise ratio comparison.

The average SNR of the textrode was 33.6 dB ± 0.14 (±0.41%) at a 95% confidence interval, which shows that the values are not significantly different. Therefore, the sensing ability of the textile-based EEG electrode is excellent. Moreover, the SNR of the textrode was higher than that of the standard wet gold cup electrodes (+4.2%), as shown in Figure 16. However, the SNR of the hook fabric textrode was slightly lower (−2.98%) than that of the dry Ag/AgCl comb electrode, indicating that the textrode still needs some improvement. This effect could be due to the fact that signals are collected from many narrower scalp contact points in the hook fabric textrode than in the dry Ag/AgCl comb electrode. In addition, the height of the hook was three-fold shorter than the length of the comb. Thus, improving the length and thickness of the hook can increase SNR.

### 3.6. Effects of Size and Bending on Signal-to-Noise Ratio

Doubling the size of the hook fabric EEG electrode (surface area of 2π cm^2^) resulted in a 17.3% increase in the signal-to-noise ratio (SNR), elevating it from 33.6 dB to 39.4 dB. This improvement can likely be attributed to the increased contact surface area and the greater number of hooks. However, it is essential to consider that enlarging the electrode size also increases its weight, which may be a concern in applications involving multiple electrodes and long-term monitoring. Furthermore, when the size was increased fourfold, the hook electrode became suspended over the hair, preventing it from making contact with the skin.

**Figure 17:**
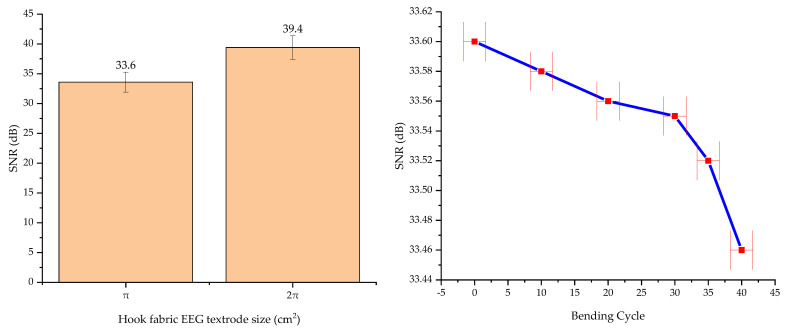
Effects of hook fabric EEG textrode size (**left**) and bending (**right**) on signal-to-noise ratio (SNR).

On the other hand, the electrodes exhibited no significant variance in SNR when subjected to up to 30 bending cycles with a 5mm radius. Analysis using a one-way ANOVA Test at a 95% confidence interval revealed that the differences in SNR mean for up to 30 bending cycles were statistically insignificant, yielding a *p*-value of 0.12. However, upon reaching 35 bending cycles, a statistically significant reduction in SNR emerged, with *p*-values of 0.02. The effects of hook fabric EEG electrode size and bending on SNR are shown in Figure 17.

### 3.7. Sustainability Concerns

Overall, the hook fabric EEG spectrum gave comparable EEG signals, and ITC, ERSP, and log PSD plots across all main EEG bandwidths. The signal-to-noise ratio was higher than that of standard wet gold cup electrodes. However, the signal-to-noise ratio was slightly lower than that of the dry Ag/AgCl comb electrode. This can be further solved by improving the hook properties, such as length and thickness. The use of a conductive polymer to explore a new type of hook fabric may boost the signal-to-noise ratio.

The raw material from which the novel hook fabric EEG electrode was constructed and its structural design were set apart from those of other commercial electrodes. Furthermore, they provide additional functions and applications. An optimized hook fabric electrode provides a sustainable benefit over commercial electrodes, as shown below.

**Avoids Conductive Gel:** At the clinical level, a conductive adhesive gel is used to bridge the EEG electrode with the skin and scalp. To apply the gel, skin preparation and scratching steps are compulsory, which in turn consumes more time. In addition, the scratching of the skin leaves skin lesions. Users may also be allergic to gel. The gel must be washed off afterward. This novel hook-fabric electrode completely avoids the use of conductive gel.

**Avoids shaving the head:** This electrode has hooks that keep it in the right area, as the hooks are entangled with the hair. The end tips of the hook are in contact with the skin and the scalp. Therefore, the EEG measurements are performed without shaving the head.

**Convenient for long-term monitoring:** Although commercial dry flat electrodes are made of metal, their weight and rigidity render them unsuitable for portable purposes. Dry flat electrodes do not work in the hairy region. Although commercial spike/comb electrodes made of metals and conductive plastics were later introduced for use in hairy areas, they are still heavy and rigid in structure, making them unsuitable for portable purposes. In addition, they can penetrate the skin and cause infections. The hook fabric electrode avoids hair having to be shaved. In addition, it is lightweight and flexible with textile softness, making it a suitable candidate for long-term monitoring for wearable purposes.

**Simple to construct and economical:** The hook fabric electrodes can be produced much more cheaply than existing dry EEG comb electrodes, i.e., ∼1.5 euro per electrode, while the hook fabric can be produced for less than 1 euro. In addition, as a textile-based electrode, the hook fabric electrodes give more freedom to easily design the desired size and shape.

**Integrable to textile structures:** Hook fabric electrodes can be easily integrated into textile structures, for example, by sewing. Therefore, electrodes are suitable for wearable purposes, where people need them for their daily activities. The electrodes are then washed.

**Versatile application:** The hook fabric electrode can be used as an EEG, ECG, or EMG electrode. They can also be used by both humans and animals. This versatile application would certainly be useful for the existing electrodes.

**Lesser environmental burden:** Because a large proportion of their constituents are textiles, with the potential to evolve from biodegradable substances and conductive polymers, the introduction of such textile-based electrodes could play a role in minimizing the burden of metallic electrode waste.

## 4. Future Prospects

The development of an optimized hook fabric textrode, combined with clinical validation and application in medical diagnostics, represents a significant step forward in the field of biopotential electrodes. The integration of conductive polymer and meticulous design optimization undoubtedly leads to superior performance and opens up new possibilities for biopotential measurements. Through dedicated research and testing, this technology can revolutionize the way we capture and utilize biopotential signals for both research and clinical applications.

**Enhancing Signal-to-Noise Ratio:** To address the current limitations, it is important to focus on enhancing the hook fabric’s properties. The first step is to conduct detailed research and experimentation to determine the ideal length and thickness of the hook fabric textrode. This process involves iterative testing and optimization to achieve the best signal quality and minimize noise interference.

**Exploring Conductive Polymer:** The integration of conductive polymer into the hook fabric holds promise for boosting the signal-to-noise ratio further. Investigating various conductive polymer materials and their compatibility with the hook fabric is important. This exploration can lead to the development of a new and improved hook fabric textrode with superior performance.

**Application in Biopotential Measurements**: Once the optimized hook fabric textrode is developed, the next phase of research involves applying it in biopotential measurements. This encompasses conducting ECG and EMG measurements on both human and animal subjects. Rigorous testing can ensure the reliability and accuracy of the measurements obtained using an improved textrode.

**Clinical Validation and Commercialization:** For successful commercialization of biopotential electrodes, a comprehensive clinical validation is necessary. A large-scale study involving a diverse group of test subjects should be conducted to assess the efficacy and safety of the optimized hook fabric textrode. The data obtained from this clinical validation are crucial in gaining regulatory approvals and building trust within the medical community.

**Clinical Diagnosis Application:** To utilize the hook fabric textrode for clinical diagnosis, further testing on patients should be carried out. These tests would focus on various medical conditions in which biopotential measurements play a vital role in diagnosis and treatment monitoring. The results of these tests could provide valuable insights into the textrode’s clinical utility and pave the way for its widespread adoption in healthcare settings.

## 5. Conclusions

Currently available EEG electrodes in the market are not suitable for wearable applications or long-term monitoring. To address these challenges, this research explored the use of textile electrodes for tracking brain activity. A textile-based EEG electrode made from silver-plated hook fabric was developed, capable of collecting high-quality EEG signals comparable to those of standard wet gold cups and dry Ag/AgCl comb electrodes. More precisely, the log power spectral density (PSD) exhibited a linear decrease as the frequency domain increased, with the maximum log PSD for all electrodes observed within the delta EEG bandwidth, ∼35 µV^2^/Hz. The hook fabric textrode eliminates the need for adhesive conductive gel and hair shaving. Moreover, it exhibited lower skin-to-electrode impedance than the dry Ag/AgCl comb electrodes and achieved a higher signal-to-noise ratio (SNR) compared to the standard wet gold cup electrode (+4.2%). Doubling the size of the hook fabric electrode results in a 17.3% increase in SNR, and this electrode maintains statistically similar SNR values for up to 30 bending cycles. Additionally, a knitted net bridge EEG cap was created and compared with universal EEG bridges. Both the hook fabric textrode and the knitted net bridge EEG cap were evaluated at the clinical level using the Brain Quick^®^ Clinical EEG Line.

## Data Availability

The data presented in this study are available on reasonable request from the corresponding author. The data are not publicly available due to privacy reasons.
